# Factors associated to inconsistent condom use among sex workers*

**DOI:** 10.1590/1518-8345.2951.3226

**Published:** 2019-12-05

**Authors:** Rosilane de Lima Brito Magalhães, Laelson Rochelle Milanês Sousa, Elucir Gir, Marli Teresinha Gimeniz Galvão, Vanessa Moura Carvalho de Oliveira, Renata Karina Reis

**Affiliations:** 1 Universidade Federal do Piauí, Departamento de Enfermagem, Teresina, PI, Brazil.; 2 Universidade de São Paulo, Escola de Enfermagem de Ribeirão Preto, PAHO/OMS Collaborating Centre for Nursing Research Development, Ribeirão Preto, SP, Brazil.; 3 Scholarship holder at the Coordenação de Aperfeiçoamento de Pessoal de Nível Superior (CAPES), Brazil.; 4 Universidade Federal do Ceará, Departamento de Enfermagem, Fortaleza, CE, Brazil.

**Keywords:** Sex Workers, Sexual Partners, Condoms, Brazil, HIV, Sexual Behavior, Profissionais do Sexo, Parceiros Sexuais, Preservativos, Brasil, HIV, Comportamento Sexual, Trabajadores Sexuales, Parejas Sexuales, Condones, Brasil, VIH, Conducta Sexual

## Abstract

**Objective:**

to analyze the factors associated to the inconsistent condom use among sex workers.

**Method:**

a transversal study, carried out in prostitution area, using the *Respondent Drive Sampling*. The sample was calculated based on the information by the Sex Workers Association: 600 female sex workers. The study selected seven women with different characteristics regarding color, age, and place of work, who were called seeds. After the participation, they received three coupons to recruit other participants in order to obtain a representative sample. The definition of inconsistent condom use was determined as occasional use or never using it. Univariate analyses and a multivariate logistic regression were performed.

**Results:**

416 female sex workers participated in the study. The associated factors were having studied for less than eight years (Odds Ratio = 27.28), not having a permanent partner (Odds Ratio = 2.79), high alcohol use (Odds Ratio = 5.07), and being black (Odds Ratio = 2.21).

**Conclusion:**

the factors associated to inconsistent condom use were: lower education levels, not having a permanent partner, high alcohol use, and being black.

## Abstract (pt)

**Objetivo:**

analisar os fatores associados ao uso inconsistente do preservativo entre trabalhadoras do sexo.

**Método:**

estudo transversal, realizado em áreas de prostituição, usou-se o *Respondent Drive Sampling*. O Cálculo amostral foi realizado com base na informação da Associação das Trabalhadoras do Sexo: 600 mulheres trabalhadoras do sexo. Foram selecionadas sete mulheres com características diferentes em relação à cor, idade e local de atuação, chamadas de sementes. Após a participação, elas receberam três cupons para recrutar outras participantes para obter uma amostra representativa. A definição de uso inconsistente do preservativo foi determinada como uso ocasional ou nunca usar. Foram realizadas análises univariadas e regressão logística multivariada.

**Resultados:**

participaram do estudo 416 mulheres trabalhadoras do sexo. Os fatores associados foram ter menos que oito anos de estudo (Odds Ratio = 27,28), não ter parceiro fixo (Odds Ratio = 2,79), uso de álcool elevado (Odds Ratio = 5,07) e cor da pele preta (Odds Ratio = 2,21).

**Conclusão:**

os fatores associados ao uso inconsistente do preservativo foram: menor escolaridade, não ter parceiro fixo, uso elevado de álcool e cor da pele preta.

## Abstract (es)

**Objetivo:**

analizar los factores asociados al uso inconsistente del preservativo entre trabajadoras del sexo.

**Método:**

estudio transversal, que ha sido realizado en áreas de prostitución, se usó el *Respondent Drive Sampling*. El Cálculo de muestra fue realizado con base en la información de la Asociación de las Trabajadoras del Sexo: 600 mujeres trabajadoras del sexo. Fueron seleccionadas a siete mujeres con características distintas en relación al color, a la edad y al local de actuación, llamadas de semillas. Después de la participación, ellas recibieron tres cupones para reclutar a otras participantes para obtener una muestra representativa. La definición de uso inconsistente del preservativo fue determinada como uso ocasional o nunca usar. Fueron realizados análisis univariados y regresión logística multivariada.

**Resultados:**

participaron del estudio 416 mujeres trabajadoras del sexo. Los factores asociados fueron tener menos que ocho años de estudio (Odds Ratio = 27,28), no tener pareja fija (Odds Ratio = 2,79), uso de alcohol elevado (Odds Ratio = 5,07) y color de la piel negro (Odds Ratio = 2,21).

**Conclusión:**

los factores asociados al uso inconsistente del preservativo fueron: menor escolaridad, no tener pareja fija, uso elevado de alcohol y color de la piel negro.

## Introduction

The infection by the human immunodeficiency viruses (HIV) remains a global challenge^(^ 1 ^)^. In the Brazilian context, higher prevalence was detected in populations that are more vulnerable to the virus, such as drug users, of 22.6%^(^ 2 ^)^, Men who have Sex with Men (MSM), of 18.4%^(^ 3 ^)^, and Sex Workers (SW), who present a variation from 1% to 4.8%^(^ 4 ^-^ 6 ^)^.

In a concentrated epidemy, populations under more risk have a fundamental role in the infection dynamics. Female sex workers are a population of high risk for HIV and are considered key-populations for the control of HIV and other Sexually Transmitted Infections (STI)^(^ 6 ^)^.

In Brazil, since 2002, sex work is an occupation recognized by the Brazilian Ministry of Work, even so, it is still needed to widen this discussion to effectively regulate the profession in he country^(^ 7 ^)^. However, these women still face difficulties in accessing health services, information on prevention, besides the lack of specific interventions.

The male condom is the main and more effective prevention mean recommended as intervention in contexts of sexual work^(^ 7 ^)^. In countries with different levels of development, income, and culture, the risk of acquiring HIV infection and other STIs is connected to the inconsistent condom use^(^ 8 ^-^ 12 ^)^.

The inconsistent condom use by sex workers is related to factors such as the criminalization of sexual work and the difficulties of negotiating with clients, besides the stigma related to the work, contributing for the difficulty in having access to condoms^(^ 13 ^-^ 14 ^)^. In this sense, behavioral interventions to increment the use of condoms among female sex workers have been described as efficient tools for the prevention of HIV and other STIs^(^ 15 ^)^.

Among the few studies on the factors associated to condom use among sex workers, a review study has shown that behavioral interventions have increased the use of condoms, but there is lack of information on other outcomes related to the use of condoms by key-populations^(^ 16 ^)^. Another review has also presented that there are evidences of the efficiency of behavioral interventions, with better results for the use of condoms with paying partners^(^ 17 ^)^.

In this view, a study has shown that, for interventions to be more effective, the vulnerability of sex workers must be understood as an occupational problem, and interventions must involve the workers themselves in the empowering process, so to have more chances of success in their daily work lives^(^ 18 ^)^.

However, there are gaps regarding wide sample that cover SW who act on the most varied places of sexual work, such as the streets, squares, night clubs, or even in specific agencies for that end. Given that, it is still necessary to develop investigations that can list heterogeneous samples aiming at an accurate understanding of the use of condoms, be it in public or private places. International experiences, such as China, indicate this difficulty due to the work being stigmatized^(^ 19 ^)^.

Therefore, given the recognition of the vulnerability of HIV and sex workers, and the special importance of the use of condoms for this population, this study aims to analyze the factors associated to the inconsistent condom use among female sex workers.

## Method

It is a transversal study^(^ 20 ^)^, performed with Sex Workers (SW) who work in prostitution areas of a capital city of the Northeast of Brazil, between March 2014 and September 2017.

The participants were recruited by the method *Respondent Drive Sampling* (RDS), which is a sampling methodology used for recruiting populations of difficult access, in which the participants themselves are responsible for recruiting other individuals of the same category as theirs.

The RDS method includes many requisites that are essential to create a representative sample and it has been recommended to improve recruiting with populations of difficult access. To meet the demands of the RDS method, seven sex workers were selected, who had different characteristics regarding color (white and not white), age (young, young adult, and elderly), and place of work (closed and open areas), which constituted the first participants and were called seeds.

Thus, each participant received three valid coupons and was oriented to invite three more sex workers, belonging to their acting network, until the obtention of a significant sample^(^ 21 ^)^. The valid coupons had very well defined information on the place and time for data gathering, which happened weekly in the mornings, in a specific place of the central area of the municipality where the collection was made.

Therefore, the following inclusion criteria were considered: being 18 years-old or older and having acted as sex workers in the municipality for at least four months. In opposition, the following exclusion criteria was chosen: being visibly under the influence of drugs, including alcohol, during the interview.

The tool used for data gathering was developed by the researchers, considering the interested variables for the investigation and the characterization of the participants, and it was validated regarding the shape and content by specialists on the theme and on the method.

The composition of the final sample was based on the verbal information by the Sex Workers Association that, in the studied municipality, there would be 600 women acting as sex workers. Besides that, it was considered the prevalence of 1.8% for HIV^(^ 5 ^)^, with tolerable risk of error of 2%, reliability index (RI) of 95%, and the addition of 10% due to eventual losses.

The data were typed twice in the Excel in an electronic spreadsheet, and, after validation, were exported to the software *Statistical Package for the Social Sciences* 21.0. To verify the relations between variables, the chi-square test was used, considering a statistical significance of p<0.005.

For the logistic regression, the dependent variable was the use of condoms, evaluated as dichotomous (Inconsistent/Consistent). The Consistent category was considered as a reference level. We highlight that the definition of inconsistent condom use was determined as using it irregularly (occasionally or never) during sexual practices (oral, anal or vaginal)^(^ 9 ^)^. The independent variables used in the study were: age group (18 to 24, 25-39, 40-59), color (white/yellow, black, brown), schooling levels (≤ 8 years of study and > 8 years of study), marital status (single, married/widow, divorced), income (no income, 1 to 2 minimum wages, 2 to 3 minimum wages, 4 to 10 minimum wages), type of sexual practice (vaginal, anal or more than 1 type), permanent partner (yes/no), use of drugs (yes/no), alcoholic drinks (never drinks, light consumption, moderate consumption, high consumption), and place of prostitution (open, closed).

For the selection of independent variables, it was used the Likelihood-ratio test, the variable with the highest p-value was removed from the model and a new adjustment was done. Based on the chosen model, the Odds Ratio (OR) were calculated, considering a reliability interval of 95% for each of the variables in the model, as well as the calculation of values associated to the inconsistent condom use according to the combination of variables in the model. All analyses were performed considering the significance level of 5% (α=0.05) and by the program R version 3.4.3.

The study was approved by the Research Ethics Committee of the Universidade Federal do Piauí, under the number 0425.0.045.000-11. We highlight that all ethical aspects of researches involving human beings were followed.

## Results

416 SW participated in the study, with an average age of 30.4 years-old, 366 (88%) had low schooling levels, 341 (82%) were single, 174 (41.8%) had a monthly income of up to two minimum wages, 179 (43%) were self-declared white, according to Table 1.

**Table 1:** Socio-demographic factors associated to consistent and inconsistent condom use among sex workers. Teresina, PI, Brazil, 2017

Variables	Total n=416	Use of male condoms		P value*
	N (%)	Consistent n=329	Inconsistent n=82	
Age group (years)				
18-24	104 (25,0)	66 (63,5)	38 (36,5)	
25-39	259 (62,3)	219 (84,6)	40 (15,4)	<0,001
> 40	53 (12,7)	44 (83,0)	9 (17,0)	
Schooling level (years)				
< 8	366 (88,0)	280 (76,5)	86 (23,5)	<0,001
≥ 8	50 (12,0)	49 (98,0)	1 (2,0)	
Marital status				
Single	341 (82,0)	269 (78,9)	72 (21,1)	
Married	22 (5,3)	16 (72,7)	6 (27,3)	0,003
Divorced	50 (12,0)	44 (88,0)	6 (12,0)	
Widow	3 (0,7)	0	3 (100,0)	
Income (minimum wage)†				
No income	21 (5,0)	16 (76,2)	5 (23,8)	
1 to 2	174 (41,8)	157 (90,2)	17 (9,8)	<0,001
2 to 3	117 (28,1)	81 (69,2)	36 (30,8)	
> 4	104 (25,0)	75 (72,1)	29 (27,9)	
Skin color				
Black	146 (35,1)	120 (82,2)	26 (17,6)	
Brown	87 (20,9)	68 (78,2)	19 (21,8)	0,045
White	179 (43,0)	140 (78,2)	39 (21,8)	
Yellow	4 (1,0)	1 (25,0)	3 (75,0)	
Origin				
Capital	323 (77,6)	259 (80,2)	64 (19,8)	0,304
Other	93 (22,4)	23 (24,7)	70 (75,3)	

* P value = Chi-square Test;

† Income (minimum wage) = The value of the minimum wage in Brazil in 2017 was R$ 937.00

Of the participants, 359 (93.5%) reported vaginal sexual practices and 82 (20%) used condoms inconsistently, according to Table 2.

**Table 2:** Factors related to sexual behavior associated to inconsistent condom use among sex workers. Teresina, PI, Brazil, 2017

Variables	Total n=416	Uso do preservativo masculino		P value*
	N (%)	Consistent n=329	Inconsistent n=82	
Sexual practice				
Anal	12 (2,9)	11 (91,7)	1 (8,3)	
Vaginal	359 (93,5)	280 (78,0)	79 (22,0)	0,335
Anal and vaginal	45 (3,6)	38 (84,4)	7 (15,6)	
Permanent partner				
Yes	131 (31,5)	120 (91,6)	11 (8,4)	<0,001
No	284 (68,3)	208 (73,2)	76 (26,8)	
Number of clients/week				
1 to 5	138 (33,2)	113 (81,9)	25 (18,1)	
6 to 10	192 (46 ,2)	147 (76,6)	45 (23,4)	0,575
11 to 15	54 (13,0)	42 (77,8)	12 (22,2)	
> 15	32 (7,7)	27 (84,4)	5 (15,0)	
Place of prostitution				
Squares	168 (40,4)	139 (82,7)	29 (17,3)	
Street	15 (3,6)	6 (40,0)	9 (60,0)	<0,001
Bars	193 (46,4)	144 (74,6)	49 (25,4)	
Night clubs	40 (9,6)	40 (100)	0	
Risk perception				
None	50 (12,0)	40 (80,0)	10 (20,0)	
Little	243 (58,4)	179 (73,7)	64 (26,3)	0,006
Big	119 (28,6)	106 (89,1)	13 (10,9)	
HIV positive+†	4 (1,0)	4 (100,0)	0	
Variables related to life habits				
Use of alcohol				
No	86 (20,7)	80 (93,0)	6 (7,0)	
Light	23 (5,5)	21 (91,3)	2 (8,7)	<0,001
Moderate	150 (36,1)	105 (70,0)	45 (30,0)	
High	157 (37,7)	123 (78,3)	24 (21,7)	
Use of illegal drugs				
Yes	237 (57,0)	175 (53,2)	62 (71,3)	0,002
No	179 (43,0)	154(46,8)	25 (28,7)	

* P value = Chi-square Test;

† HIV = Human Immunodeficiency Virus

The variables schooling levels, ≤8 years of study (OR=27.28; RI95%:3.45 - 215.47), not having a permanent partner (OR = 2.79; RI95%:1.5 - 5.2), high use of alcohol (OR = 5.07; RI95%:1.87 - 13.74), and black skin color (OR = 2.21; RI95%:1.17 - 4.18) have been associated to the inconsistent male condom use (Table 3).

**Table 3:** Result of the logistic regression analysis: independent variables associated to inconsistent male condom use among female sex workers. Teresina, PI, Brazil, 2017

Variable	Gross Odds Ratio [95% RI*]	P value†	Adjusted Odds Ratio [95% RI*]	P value†
Schooling levels (years)				
≤8	15.05 (2.05,110.59)	0,008	27.28 (3.45,215.47)	0,002
Income (minimum wage)‡				
No income	0.81 (0.27,2.41)	0,702	0.44 (0.12,1.62)	0,217
One to two wages	0.28 (0.14,0.54)	<0,001	0.23 (0.11,0.49)	<0,001
Two to three wages	1.15 (0.64,2.06)	0,639	0.72 (0.37,1.43)	0,353
Permanent partner				
Yes				
No	3.19 (1.83,5.54)	<0,001	2.79 (1.5,5.2)	<0,001
Use of alcohol				
High use	3.69 (1.48,9.18)	0,005	5.07 (1.87,13.74)	<0,001
Moderate use	5.71 (2.32,14.06)	<0,001	8.2 (3.04,22.14)	<0,001
Light use	1.27 (0.24,6.75)	0,779	2.17 (0.35,13.24)	0,403
Marital status				
Divorced	0.24 (0.07,0.79)	0,019	0.16 (0.04,0.62)	0,008
Single	0.48 (0.2,1.12)	0,089	0.51 (0.18,1.44)	0,202
Skin color				
Brown	1.48 (0.78,2.83)	0,231	1.89 (0.86,4.13)	0,112
Black	1.94 (1.13,3.32)	0,017	2.21 (1.17,4.18)	0,014

* RI = Reliability Interval;

† P value = Chi-square Test;

‡ Income (minimum wage) = The value of minimum wage in Brazil in 2017 was R$ 937.00

## Discussion

In this study, the main factors associated to inconsistent condom use among sex workers were identified: schooling level, not having a permanent partner, high use of alcohol, and black skin color.

The limitations of the study are related to the difficulty in accessing the interested population, due to their work characteristics and places of action. Thus, the RDS technique was used, which can lead to a selection bias in the recruitment of participants, since sex workers who are distant from the networks of the recruited participants tend to be out of the sample.

It is vital for further studies to overcome such limitations, broadening the recruitment through searches in social networks, dating sites, escort sites, and geolocation apps. These are strategies that may overcome the indicated limitations.

The inconsistent condom use among sex workers has been reported by Brazilian researchers^(^ 21 ^)^. In this sense, this study reflects the need for more attention to similar populations and for the implementation of interventions that aim to encourage the use of combined preventive methods, considering that the inconsistent condom use is high among this population.

In this sense, international evidences corroborate the previous affirmations: a systematic review on sex workers in Uganda has shown that 33.3% to 55,1% of them reported inconsistent condom use^(^ 22 ^)^.

Besides, other international studies have reported higher rates: 34.5% in Ukraine, and 76.8% in Uganda^(^ 9 ^,^ 23 ^)^. The inconsistent condom use among women acting on sexual work is a concerning data. Interventions such as health education must be implemented to expose information on the benefits of using condoms, as well as providing guidance on the proper ways of use, contributing for the incentive to the regular adhesion to condoms.

In relation to the factors associated to the inconsistent use, it was identified that schooling levels among the investigated population have been similar to national researches, lower than 8 years of study. A research performed in the central region of Brazil has shown that 54.5% of sex workers has studied from 5 to 9 years^(^ 5 ^)^.

Within this scenario, evidences indicate that the use of condoms is associated to schooling levels. The inconsistent use was associated to low schooling levels among people attended by the primary care in South Africa^(^ 24 ^)^. Besides that, there is a strong documented association between low schooling levels and unprotected sexual relations^(^ 25 ^)^.

Therefore, low schooling levels and the inconsistent condom use imply on the risk of being exposed to HIV. A similar result was observed in a study with men who have sex with men, in China^(^ 26 ^)^. Moreover, women with Higher Education had less probability of being infected^(^ 27 ^)^.

Brazilian studies regarding intervention on this issue are needed. There are evidences on the efficiency of behavioral interventions that lead to positive results in the adhesion to consistent condom use among sex workers^(^ 17 ^)^.

In this perspective, the equal access to education is needed both for female sex workers and for other HIV vulnerable populations, given that the proper prevention involves multiple factors, including the need for access to information on STI/HIV preventive strategies, understanding it and incorporating it in their prevention and care repertoires.

Other variables were also associated to the inconsistent use: not having a permanent partner, high alcohol use, and black skin color. A similar result was observed among sex workers in Uganda with regular clients^(^ 9 ^)^. It is inferred that female sex workers who have a permanent partner present higher motivation to protect themselves and, thus, also protect their partners. Regarding the use of alcoholic drinks, studies performed in Ukraine and Uganda corroborate the findings in this research^(^ 9 ^,^ 23 ^)^.

It is known that the alcohol consumption among sex workers eases the risk for HIV infection, specially when the use happens during the sexual interaction with clients^(^ 28 ^)^. Alcohol consumption is part of the social interaction for the most diverse populations around the world and it is inserted in celebration environments^(^ 29 ^)^. However, its high consumption may affect the health^(^ 30 ^)^ and, in the context of sexual work, it may be an important aspect for the vulnerability to HIV and other health conditions.

Indeed, the use of alcohol has been described as a factor that, besides interfering with the overall health state, had impacted on HIV cases. Evidences show that the use of alcohol presents reflexes on the amount of diseases and mortality, in many African countries, directly impacting on the incidence of course of HIV/AIDS^(^ 31 ^-^ 32 ^)^. It may also interfere with the cognitive control and reduce the perception of vulnerability, contributing for higher exposition to HIV^(^ 33 ^)^.

## Conclusion

This study contributes to the knowledge on the use of condoms by sex workers, indicating that it was used irregularly. Therefore, the results have evinced the factors related to the inconsistent male condom use, namely: having studied for less than 8 years; not having a permanent partner; high alcohol use; and black skin color.
